# Proteomics of epicardial adipose tissue in patients with heart failure

**DOI:** 10.1111/jcmm.14758

**Published:** 2019-10-31

**Authors:** Lei Zhao, Zongsheng Guo, Pan Wang, Meili Zheng, Xiaoyan Yang, Ye Liu, Zheng Ma, Mulei Chen, Xinchun Yang

**Affiliations:** ^1^ Heart Center & Beijing Key Laboratory of Hypertension Beijing Chaoyang Hospital Capital Medical University Beijing China

**Keywords:** epicardial adipose tissue, heart failure, proteomics

## Abstract

Epicardial adipose tissue (EAT) is a metabolically active visceral fat depot closely linked to the pathogenesis of heart failure (HF). But the molecular signatures related to the mechanism of HF have not been systematically explored. Here, we present comprehensive proteomic analysis of EAT in HF patients and non‐HF patients as controls. A total of 771 proteins were identified in liquid chromatography‐tandem mass spectrometry experiments. Amongst them, 17 increased in abundance in HF and seven decreased. They were involved in HF‐related processes including inflammation and oxidative stress response and lipid metabolism. Of these proteins, serine proteinase inhibitor A3 (Serpina3) levels in EAT were highly up‐regulated in HF, with HF/non‐HF ratio of 4.63 (*P* = .0047). Gene expression of Serpina3 via quantitative polymerase chain reaction was significantly increased in the HF group. ELISA analysis confirmed a significant increase in circulating plasma Serpina3 levels in the HF group (*P* = .004). In summary, for the first time, we describe that parts of EAT proteome may be reactive and work as modulators of HF. Our profiling provides a comprehensive basis for linking EAT with pathogenesis of HF. Understanding the role of EAT may offer new insights into the treatment of HF.

## 1 INTRODUCTION

Heart failure (HF) is a major healthcare issue affecting approximately 3% of the general population.^1^ With the increasing aged population, it has become the main cause of mortality and hospitalization. The pathogenesis of HF is multifactorial, and the mechanisms are not yet fully elucidated. This is a consistent body of evidence demonstrating that patients with HF are burdened by metabolic comorbidities, including dysfunction of lipid metabolism, that dramatically impact long‐term outcomes.^2^, ^3^ Recently, adipose tissue has been increasingly realized to contribute to this pathophysiological process.

Adipose tissue, identified as a complex endocrine organ, has profound effects on the cardiovascular system by exerting a wide range of regulatory functions, which is classified into two anatomically distinct types, subcutaneous and visceral adipose tissue.^4^, ^5^ Those fat depots surrounding the heart, also referred to as epicardial adipose tissue (EAT), function as metabolic transducers in the regulation of cardiac functions in HF.^6^, ^7^ EAT is a metabolically active visceral fat depot located in atrioventricular and interventricular grooves. There is no anatomical boundary between EAT and myocardium, and they share the coronary microcirculation. Due to this anatomical arrangement, there are potential physiological (including vasocrine and paracrine) interactions between these two tissues.^5^, ^8^ EAT serves as a local energy source during high‐energy demands by releasing fatty acids to the myocardium and protecting the heart against high fatty acid levels and associated lipotoxicity. The relationship between EAT thickness/volume and the extent of cardiovascular disease has already been established.^9^, ^10^, ^11^, ^12^ The accumulation of EAT has been identified to be a significant feature of chronic metabolic status related to HF. In clinical setting, HF patients had more EAT compared with controls and EAT volume was closely associated with the presence of atrial fibrillation and type 2 diabetes mellitus and with biomarkers related to myocardial injury.^13^

As the proteome is the determinant of the cell and can better elucidate the process behind the manifestation of diseases, the label‐free quantitative proteomic approach was employed to calculate the changes in protein abundance between groups. In the proteomic study of EAT by Salgado‐Somoza and colleagues, proteins related to oxidative stress seemed to be more expressed in EAT rather than subcutaneous adipose tissue in patients with coronary artery disease, which suggested that EAT in patients with cardiovascular disease was closely associated with myocardial stress.^14^ Recently, a study on proteomics of the epicardial fat secretome has suggested EAT secretome as a possible substrate for post‐operative atrial fibrillation.^15^ However, few studies have focused on the proteomic changes of EAT in patients accompanied by HF. Therefore, our aim was to evaluate the EAT proteome and investigate substrate changes that may contribute to the genesis of HF. The use of nano‐LC‐MS/MS generated a comprehensive proteome of EAT, including 8031 peptides of 1430 proteins. From amongst them, we selected 24 differently expressed proteins in EAT that are possibly involved in HF‐related pathology. Of these proteins, serine proteinase inhibitor A3 (Serpina3) was significantly increased in EAT from HF patients, which was further confirmed in the following validation stage. These data indicate that Serpina3 may play an important role in the progression of HF.

## 2 MATERIALS AND METHODS

### 2.1 Experimental design and statistical rationale

**Figure 1:**
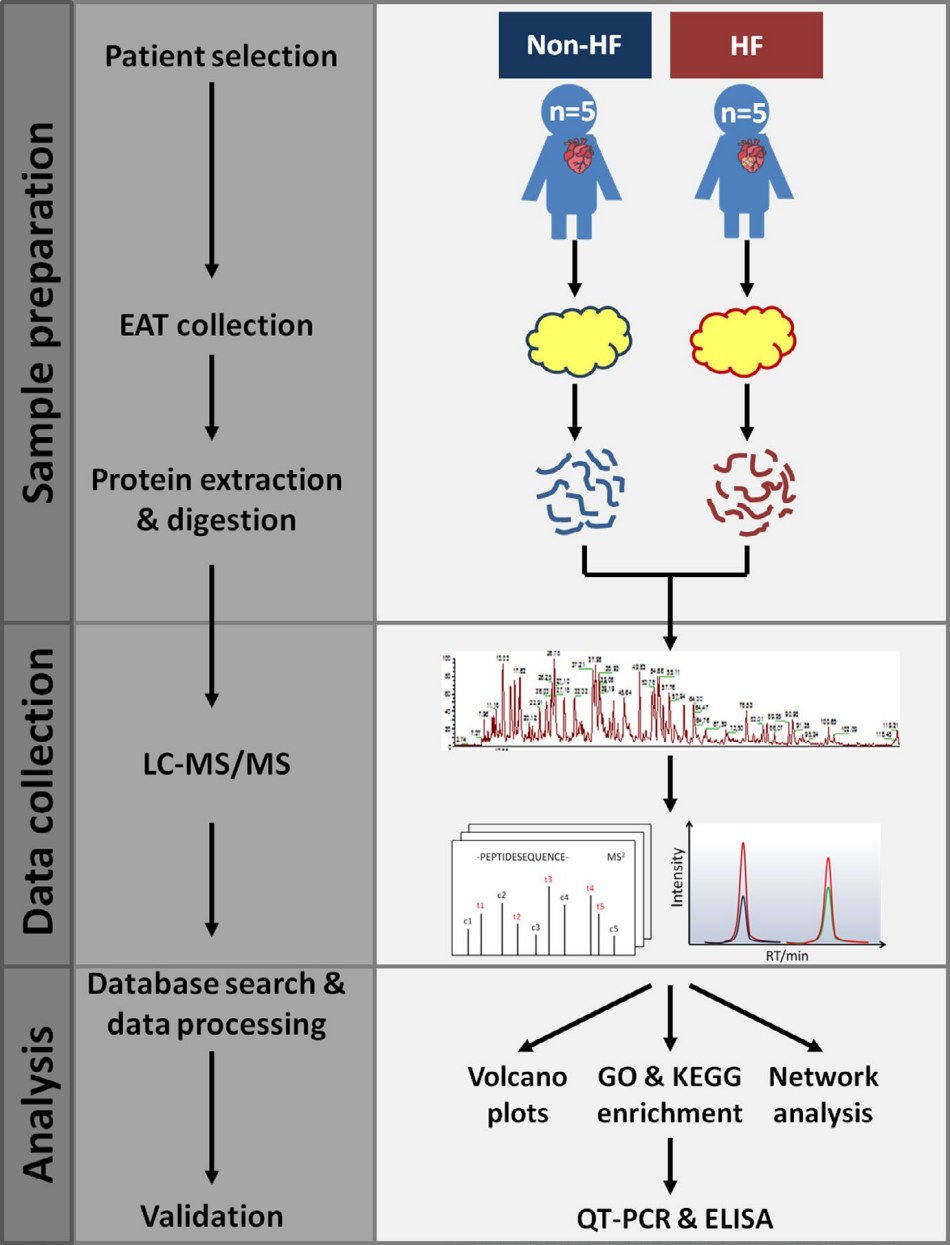
Experimental workflow of quantitative proteomic analysis of human epicardial adipose tissue using LC‐MS/MS‐based approach

In order to make comparative quantitative proteomic analyses of human EAT associated with HF, we selected patients with HF (n = 5) and patients with non‐HF (n = 5) as the control group. Enrolled patients in this study were all free of cancer. The baseline demographic and laboratory characteristics are summarized in Table S1. In HF group, four patients underwent coronary artery bypass grafting (CABG) and one patient underwent cardiac surgery for valve replacement, similar to non‐HF group. As shown in Figure 1, after collection, EAT samples were processed and analysed in a label‐free quantitative proteomic workflow. In each cohort, an equal concentration of 100‐μg proteins was isolated from EAT, proteolytically digested with trypsin and subsequently analysed using nano‐LC‐MS/MS with a nano‐UPLC system and a quadrupole‐Orbitrap mass spectrometer. The relative label‐free quantitative (LFQ) intensities of the proteins across the individual samples were acquired with MaxQuant software. The correlation coefficient for the LFQ intensities between LC‐MS/MS runs was greater than 0.80. The relative label‐free quantitation profiling was highly reproducible between two intra‐cohort or inter‐cohort LC‐MS/MS runs. Open‐source public databases were then applied in the quantitative proteomic analyses and the proteins of interest would be further verified in the following procedures. In addition to the proteomic analyses, we explored the relationships amongst clinical variables and the underlying biological processes derived from protein data.

### 2.2 Patients and tissue sampling

Epicardial adipose tissue were collected from ten patients undergoing CABG or cardiac surgery for valve replacement between April 2018 and August 2018. All EAT samples were taken under the same haemodynamic conditions, a few minutes before the extracorporeal circulation was started. EAT (volume 1‐2 cm^3^) were collected from the left‐interventricular groove, cut into small pieces, washed with ice‐cold PBS three times to remove the blood, transferred into frozen tubes, frozen in liquid nitrogen for 10 minutes and finally stored at −80°C until analysis. To confirm the validity of the selected proteins by proteomics analysis, we included patients who were diagnosed with AMI between August 2018 and February 2019 and divided into HF and non‐HF groups. The diagnosis of HF was based on symptoms and clinical examinations [brain natriuretic peptide (BNP) > 500 ng/L, enlarged left ventricular end diastolic diameter and reduced left ventricular ejection fraction (<50%)]. Baseline demographic characteristics and clinical parameters were obtained at admission. Each patient provided written informed consent after individual explanation, and the research was conducted in compliance with the principles specified for research on patients in the Declaration of Helsinki and the research protocol was approved by the ethics committee of Beijing Chaoyang Hospital.

### 2.3 Extraction and digestion of epicardial adipose proteins

Proteins were extracted from EAT using RadioImmuno Precipitation Assay buffer (Solarbio, Beijing) supplemented with protease inhibitors (Sigma‐Aldrich, St. Louis). Protein concentration was determined with BCA protein assay (Pierce, IL). Take 100‐μg proteins and add pre‐chilled acetone to alkylated protein. Extracted proteins were solubilized in 100 mmol/L ammonium bicarbonate with 1% sodium deoxycholate (SDC) and reduced with 5 mmol/L tris 2‐carboxyethyl phosphine hydrochloride for 10 minutes at 55°C, followed by alkylation with 10 mmol/L iodoacetamide for 15 minutes in the dark at room temperature. The concentration was then digested overnight at 37°C with sequencing grade modified trypsin (trypsin 1:50 protein w/w ratio; Promega, Madison, WI). After clean‐up of SDC with 2% TFA, the tryptic peptides were desalted using C18 cartridge (3M, St. Paul) and the eluted peptides were dried in a vacuum concentrator.

### 2.4 Label‐free quantitative proteomics by LC‐MS/MS

Extracted peptides were separated using a reversed‐phase column (100 μm × 150 mm, 3 μm ReproSil‐Pur 120 C18‐AQ, 1.9 μm, Dr Math) at a 300 nL/min flow rate and analysed with the EASY‐nLC nano‐UPLC (Thermo Fisher Scientific, Bremen, Germany). The UPLC mobile phase A [0.1% formic acid (FA) with 2% acetonitrile (ACN)] and B [80% ACN with 0.1% FA] were used to perform a 120‐min gradient elution as follows: 8%‐30% B for 92 minutes, 30%‐40% B for 20 minutes, 40%‐100% B for 2 minutes, 100% B for 2 minutes, 100%‐2% B for 2 minutes and finally 2% B for 2 minutes. Peptides were analysed using a Q‐Extractive mass spectrometer (Thermo Fish Scientific, Bremen, Germany) coupled with MS for the 20 highest‐intensity ions with a normalized collision energy of 27% for HCD and an isolation window of 2 m/z. The full mass and subsequent MS/MS analyses were performed in an Orbitrap analyzer with a resolution of 70 000 for MS1 (at 200 m/z) and 17 500 for MS2, respectively. The automatic gain control target for MS1 was set to 3.0 E^+6^ with max IT 50 ms and 5.0 E^+4^ for MS2 with max IT 100 ms. The top 20 most intense ions were fragmented by HCD with normalized collision energy of 27% and isolation window of 2 m/z. The dynamic exclusion was set at 30 seconds.

### 2.5 Bioinformatics and data analysis

Raw data were processed with MaxQuant software (version 1.5.6.0). The following parameters were used: carbamidomethyl [C] as fixed modification, oxidation [M] and acetyl [protein N‐term] as variable modifications, three missed cleavages were allowed. The false discovery rates (FDRs) of the peptide‐spectra matches determined by a decoy database (Uniprot_human_2016_09) search were set less than 0.01. Only unique & razor peptides were used for quantification. All the other parameters were reserved as default. Proteins were considered to be successfully identified if they could be established at greater than 99% probability with at two correct assigned peptides obtained. Protein abundance was calculated on the basis of the normalized spectral protein intensity (LFQ intensity). The significance of Log_2_LFQ intensity of proteins between HF and non‐HF group was calculated using Perseus program (Figure S1). Scatter do plots were generated, and parameters were statistically analysed using unpaired student's *t* test. *P* value < .05 was considered significant. The intracellular pathway analysis was performed using clusterProfiler in R package to search Gene Ontology (GO) and Kyoto Encyclopedia of Genes and Genomes (KEGG) database. For protein‐protein interaction network analysis, the UniProt functional annotations were used to hierarchical clustering the proteins into several clusters. Based on the raw data from MS, proteins matched in clusters were extracted and submitted to STRING database to define the physical and functional interactions amongst these proteins and then interpreted by Cytoscape into visualized network.

### 2.6 Quantitative polymerase chain reaction

Total RNA was extracted from human EAT using TRIzol reagent (Invitrogen), and RNA concentration was measured using of NanoDrop 1000 spectrophotometer (Thermo Scientific, DE, USA). Approximately 1 μg of RNA from each sample was reversely transcribed using SuperScriptTM III Reverse Transcriptase (Invitrogen) for cDNA synthesis, and Roche FastStart Universal SYBR Green Master Mix was used to perform quantitative real‐time PCR. β‐Actin was used as an endogenous control.

### 2.7 Determination of Serpina3 levels in plasma

Plasma concentrations of Serpina3 were determined by commercially available microplate ELISA kit (AACT Human SimpleStep ELISA Kit, Abcam, USA) according to the manufacturer's instructions. Venous blood samples were collected in potassium EDTA‐containing tubes. After collection, the blood sample was centrifuged immediately at 1500 *g* for 15 minutes at ambient temperature. Plasma was extracted and frozen in aliquots at −80°C until analysis. During measurement, the plasma samples were diluted 1:50 000. The intra‐assay and inter‐assay coefficients of variation were both <5%.

### 2.8 Statistics

Categorical variables were expressed as frequencies and percentages and continuous variables as the mean ± standard deviation or median (interquartile range), where appropriate. Comparisons between groups were performed using independent‐sample *t* test, Kruskal‐Wallis or Mann‐Whitney *U* tests for continuous variables and Chi‐square or Fisher's exact tests for categorical variables. Linear regression analysis was performed to calculate clinical relevant factors related to Serpina3 levels. A receiver operator characteristics (ROC) curve and the area under the curve (AUC) for the prediction of HF were analysed. All analyses were performed with IBM SPSS Statistics 24.0 (IBM Corp, Armonk, NY), and a 2‐tailed *P* < .05 was considered statistically significant.

## 3 RESULTS

### 3.1 Human EAT proteome profile associated with HF

**Figure 2:**
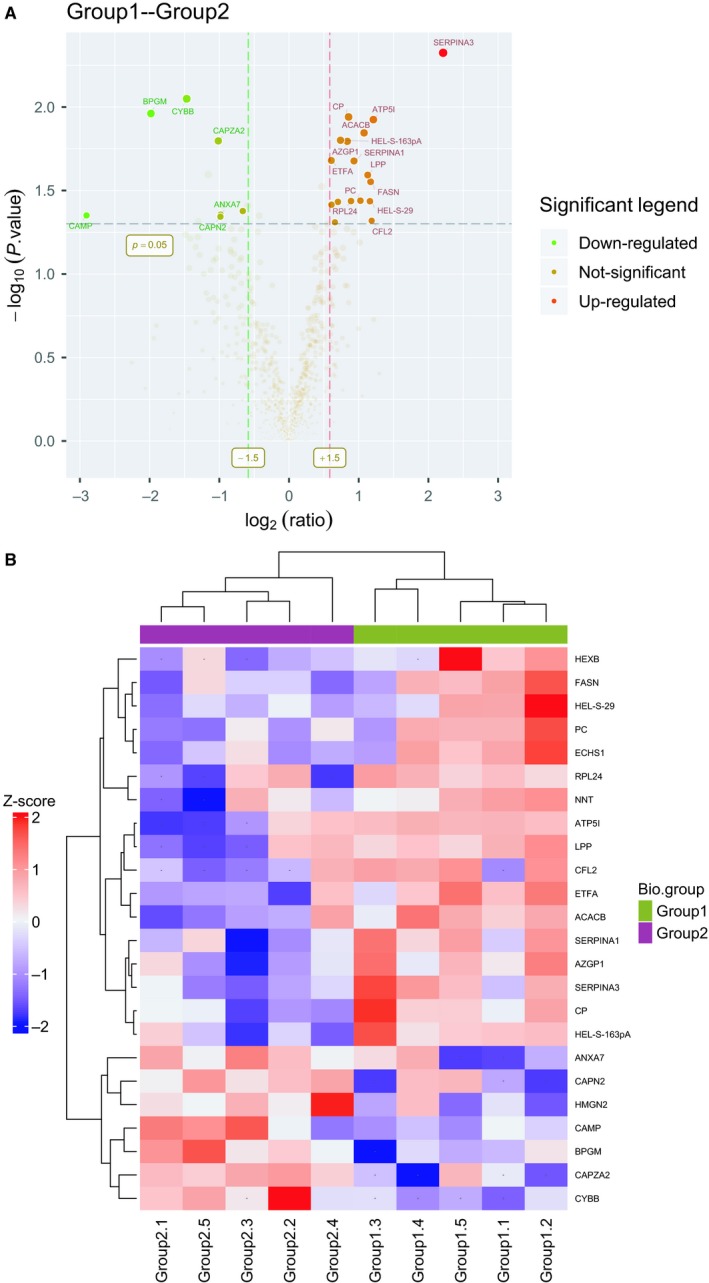
Comprehensive proteome profile of human epicardial adipose tissue. A, Significant proteins described by volcano/fold‐change plot. B, Sample cluster heat map performed with hierarchical clustering

In order to identify EAT proteins’ abundances in HF, we threw the raw data to LC‐MS/MS database for searching and got a core set of 771 quantified proteins (FDR < 0.01) (Table S3). Based on the protein ratios of the 771 EAT proteins, we then identified 24 potential differently expressed proteins in EAT associated with HF. Of the 24 proteins, 17 proteins increased in HF relative to non‐HF, whereas seven proteins decreased in HF. These proteins, especially associated with inflammation and oxidative stress response, were differently expressed between groups. Amongst these proteins, Serpina3 was significantly up‐regulated in the HF group (*P* = .0047). Detailed protein information is provided in Table 1. The distributions of statistical significance and magnitude of change for these proteins for each group are presented in volcano plots (Figure 2A). The cluster heat map, using hierarchical analysis with a Pearson correlation, separated groups with the presence of HF, and was detailed in Figure 2B.

**Table 1:** Significantly changed proteins in EAT identified by LC‐MS/MS (HF vs non‐HF) (*P* < .05)

Protein name	UniProt ID	Number of unique peptides	% sequence coverage	*P* value	Fold change (HF: non‐HF)
Alpha‐1‐antichymotrypsin	AACT_HUMAN	11	26.5%	.0047	4.63
ATP synthase subunit e, mitochondrial	ATP5I_HUMAN	2	30.4%	.012	2.31
Cofilin‐2	COF2_HUMAN	3	34.9%	.048	2.28
Fatty acid synthase	FAS_HUMAN	63	28.4%	.028	2.25
Creatine kinase B‐type	KCRB_HUMAN	7	25.2%	.037	2.24
Lipoma‐preferred partner	LPP_HUMAN	3	8.2%	.026	2.19
Acetyl‐CoA carboxylase 2	ACACB_HUMAN	20	10.3%	.014	2.11
Pyruvate carboxylase, mitochondrial	PYC_HUMAN	17	17.1%	.036	2.03
Alpha‐1‐antitrypsin	A1AT_HUMAN	16	54.3%	.021	1.91
60S ribosomal protein L24	RL24_HUMAN	2	13.2%	.037	1.85
Ceruloplasmin	CERU_HUMAN	20	25.5%	.011	1.81
Zinc‐alpha‐2‐glycoprotein	ZA2G_HUMAN	10	41.6%	.016	1.79
Alpha‐1B‐glycoprotein	A1BG_HUMAN	9	21.4%	.016	1.67
Beta‐hexosaminidase subunit beta	HEXB_HUMAN	4	8.1%	.04	1.63
NAD (P) transhydrogenase, mitochodrial	NNTM_HUMAN	4	3.9%	.049	1.58
Enoyl‐CoA hydratase, mitochondrial	ECHM_HUMAN	10	43.8%	.038	1.53
Electron transfer flavoprotein subunit alpha, mitochondrial	ETFA_HUMAN	8	38.6%	.021	1.52
Non‐histone chromosomal protein HMG‐17	HMGN2_HUMAN	2	23.3%	.042	0.63
Annexin A7	ANXA7_HUMAN	4	13.4%	.044	0.51
Calpain‐2 catalytic subunit	CAN2_HUMAN	3	5.5%	.045	0.51
F‐actin‐capping protein subunit alpha‐2	CAZA2_HUMAN	2	11.5%	.016	0.49
Cytochrome b‐245 heavy chain	CY24B_HUMAN	2	6.9%	.009	0.36
Bisphosphoglycerate mutase	PMGE_HUMAN	7	27%	.011	0.25
Cathelicidin antimicrobial peptide	CAMP_HUMAN	3	16.8%	.044	0.13

Note

UniProt ID, protein identifier within Universal Protein Resource (UniProt) knowledgebase; Number of unique peptides, peptides on which protein identification was based; % sequence coverage, percentage of sequence of the full‐length protein covered by the unique peptides that were identified.

Abbreviations: EAT, epicardial adipose tissue; HF, heart failure.

### 3.2 Functional analyses of the differential EAT proteome associated with HF

**Figure 3:**
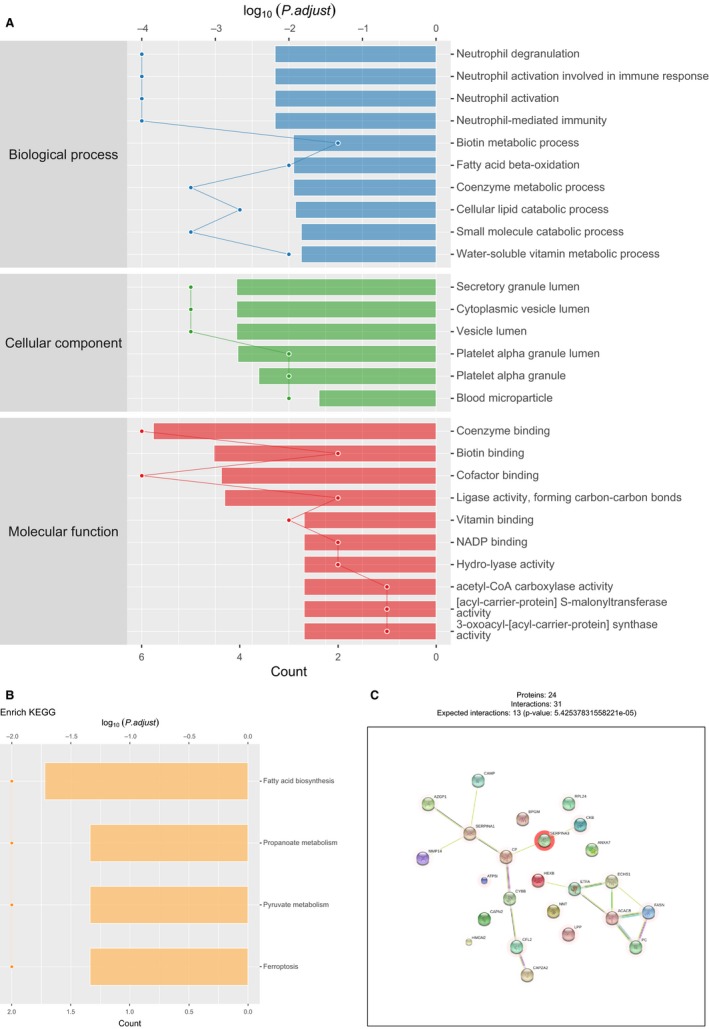
Network models describing cellular process mediated by HF‐associated EAT proteins. A, B, Gene Ontology and KEGG enrichment analysis of the biological process in the clusters of regulated proteins. C, Network models describing the up‐regulated and down‐regulated proteins. EAT, epicardial adipose tissue

We then compared differentially expressed proteins between study groups by performing enrichment analysis of Gene Ontology biological process and identified key biological processes and potential pathways that might discriminate HF from control. The cellular processes significantly represented by the EAT proteome included neutrophil activation in immune response and degranulation, biotin metabolic process, fatty acid beta‐oxidation, cellular lipid catabolic process and coenzyme and water‐soluble vitamin metabolic process, which mainly related to responses to reactive oxygen species and oxidation stress, inflammatory/immune responses and lipid metabolism. The enrichment analysis of GO cellular components revealed that most of these proteins were localized in the cytoplasm, vesicle, platelet alpha granule and blood microparticle. Through paracrine and autocrine secretion, these active factors participated in fatty acid biosynthesis, propanoate and pyruvate metabolism and ferroptosis, as described in KEGG pathway mapping (Figure 3A,B). Proteins that participate in cytoskeletal functions were down‐regulated.

### 3.3 Network analysis identifies HF‐related specific protein network in EAT

To explore the relationships amongst the differentially expressed proteins related to HF, we conducted adipose gene network analysis to identify HF‐related specific protein and extracted the subnetworks that were influenced by advanced cardiac function. For the inflammation‐related process in the network model, one notable network included Serpina3, CKB, CP, Serpina1, AZGP1, MMP14 and CAMP, which were involved in the inflammation‐related modules, mitochondrial metabolism and cell degranulation in the regulation of acute phase response. On the other hand, regarding the metabolism regulation in the constructed model, protein products of key candidates associated with lipid metabolism and fatty acid synthesis, including ACACB, FASN, ETFA, ECHS1 and PC, response significantly increased in EAT of patients with HF. Our analysis illustrates an up‐regulation in proteins and gene networks associated with leukocyte activation and macrophage activation and an increase in proteins related with fatty acid metabolism in patients with poor cardiac function, which indicating a complex relationship between cardiovascular system and adipose tissue.

### 3.4 Validation of the selected protein

**Figure 4:**
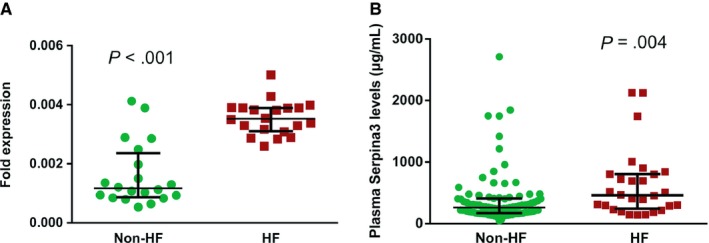
Validation of the Serpina3 expression. A, QT‐PCR validation on EAT showing significant Serpina3 gene up‐regulated in HF (*P* < .001). B, Plasma Serpina3 levels were significantly elevated in HF (*P* = .004). Error bars represent median with interquartile range. EAT, epicardial adipose tissue

In the discovery phase using label‐free LC‐MS/MS, 24 candidate EAT proteins were identified as primary targets for further clinical validation in larger cohorts. As shown in Table 1, Serpina3 levels in EAT were highly up‐regulated in HF, with HF/non‐HF ratio of 4.63 and a *P* value of 0.0047. Serpina3, also called alpha‐1 antichymotrypsin or AACT, was first discovered as an acute phase plasma protease inhibitor, which has been implicated in the pathology of complex human disorders. Gene expression levels were further confirmed via QT‐PCR on EAT samples of controls and HF (20 vs 20). The analysis confirmed a significant increase in Serpina3 in HF (*P* < .001) as shown in Figure 4A. Additionally, the circulating levels of Seprina3 were measured in patients with (n = 28) or without HF (n = 88) at admission. The basic and laboratory parameters are detailed in Table S2. As expected, patients with HF showed elevated Serpina3 levels in plasma compared with non‐HF cohort (460.08 [243.47, 803.19] vs 263.58 [171.70, 408.73] μg/mL, *P* = .004, Figure 4B). During the process of HF, many other biochemical factors and cardiac hormones are up‐ or down‐regulated, such as brain natriuretic peptide (BNP), as a feedback control for the deterioration of cardiac function. Accordingly, the relationships between baseline Serpina3 and other serum biomarkers sampled at baseline were analysed. Plasma Serpina3 concentrations correlated positively with C‐reactive protein (CRP) (*r* = 0.206, *P* = .038), erythrocyte sedimentation rate (ESR) (*r* = 0.363, *P* < .001) and BNP (*r* = 0.222, *P* = .018), whilst correlated negatively with high‐density lipoprotein (HDL) (*r* = −0.186, *P = *.047) and serum albumin (*r* = −0.201, *P = *.032). In the multivariate linear regression analysis, BMI (standardized *β* = −0.243, *P* = .032), ESR (standardized *β* = 0.269 *P* = .04) and fibrinogen (standardized *β* = 335, *P* = .012) were independent determinants of Serpina3 (Table S4). Although Serpina3 was not the best predictor of HF compared with BNP and heart rate (Table S5), the presence of increased Serpina3 levels improved the diagnosis of HF. In the ROC analysis for the prediction of HF, the AUC for BNP was 0.673 (CI: 0.549, 0.796, *P* = .006), whilst the AUC for the combination of BNP and Serpina3 was 0.727 (CI: 0.613, 0.841, *P* < .001) (Figure S2). Collectively, Serpina3 protein may play a key role in the biology of EAT, participating in the physiopathology of cardiac dysfunction.

## 4 DISCUSSION

Epicardial adipose tissue is a metabolically active visceral fat depot located in atrioventricular and interventricular grooves.^12^ Under physiological conditions, EAT can serve as a buffer, absorbing fatty acids as a lipid storage depot and as brown fat, defending the myocardium against load pressure.^6^ But in pathological conditions, EAT may experienc “phenotypic” transformation and the EAT store and metabolic activities are different from normal condition.^6^ As reported, increased volume and inflammation of EAT are associated with the progression of cardiac dysfunction in obese individual.^16^, ^17^, ^18^ However, the alterations of metabolic and functional alterations in EAT have been considered clinically important but are yet to be systematically investigated. Accordingly, in this study, we described comprehensive proteome profiles of EAT to identify the proteins possibly modulating the primary pathophysiological process, inflammatory response and metabolic alterations linked to HF. To achieve this goal, we conducted a workflow that involved a comprehensive proteome profiling of whole EAT using nano‐LC‐MS/MS, identified differently expressed proteins in EAT between HF and controls, constructed HF‐related network models and analysed biological functions of the proteome related to HF, and validated the selected protein using QT‐PCR and ELISA in a larger cohort. Using this workflow, we identified an array of 24 differentially expressed EAT proteins related to HF, participating in the prominent perturbation of inflammatory, mitochondrial and lipid metabolism pathways.

Systemic inflammatory response and oxidative stress are implicated in the deterioration of cardiac function.^15^ The inflammation‐related network in response to HF included up‐regulated Serpina3, CKB, CP, Serpina1, AZGP1 and MMP14. These have been a consensus that inflammatory status in EAT underlines the deterioration of cardiac function.^13^, ^19^, ^20^ The recruitment of proinflammatory immunocytes to EAT and releasing of proinflammatory cytokines have been implicated in the link between EAT and cardiovascular disorders. In addition, the modules in lipid metabolism and fatty acid synthesis, including ACACB, FASN, ETFA, ECHS1 and PC, responded significantly increased in the status of HF. Our data provided a detailed list of proteins in EAT potentially participated in the pathogenesis of HF, thus extending extensively the current understanding of mechanism of cardiac dysfunction. Additionally, the network model lays a foundation for combing inflammation of EAT and metabolic dysfunction in EAT with cardiovascular diseases. The network suggested that inflammation of adipose tissue surrounding heart and fatty acid oxidation should be part of pathophysiology in HF. However, as our study is mainly relied on curated databases for protein‐protein interaction, future researches are needed to investigate the exact mechanisms underlying the association of candidate EAT proteins in HF.

Amongst the differentially expressed proteins, we believe that Serpina3 make a prominent link between EAT and HF. Serpina3, also called alpha‐1 antichymotrypsin or AACT, was first discovered as an acute phase plasma protease inhibitor.^21^, ^22^ It serves the function predominantly via the regulation of neutrophil cathepsin G, leukocyte elastase and mast cell chymases. Of note, cathepsin G is contained in neutrophil granules and released as a response to inflammatory status, but the excessive release or long‐time activation would eventually result in an adverse effect. It has been implicated that Serpina3 partly contributes to the pathology of devastating human diseases, including chronic obstructive pulmonary disease, Alzheimer's disease, Parkinson's disease and cerebral haemorrhage.^21^, ^23^ However, continuous infusion of recombinant human alpha‐1 antichymotrypsin significantly attenuated myocardial ischaemia/reperfusion injury by inhibiting neutrophil‐accumulation into ischaemic‐reperfused myocardium and by inactivating cytotoxic metabolites released from neutrophils.^24^ The plasma alpha‐1 antichymotrypsin levels are described as elevated in patients with HF,^25^ as shown in our study, but the role of Serpina3 in this status, that is helping control inflammation or contributing to the deterioration of cardiac function, needs to be further illustrated. HF is a complex syndrome of inflammation, characterized by the explosion of a variety of cytokines. It has been proposed that EAT inflammation in response to metabolic disturbance leads to cardiac dysfunction via release of free fatty acid that activates the immune system and leads to release of cytokines, such as IL‐1β, TNFα, IL‐6.^26^ Importantly, human EAT obtained from obese patients with preserved ejection fraction showed a significant increase in EAT inflammation and activated M1‐phenotype macrophages.^6^, ^27^ In our study, we did not detect disturbance in immune system and cytokine cascade in plasma by either gene enrichment analysis or blood chemical examinations, but higher Serpina3 levels were closely associated with higher BNP levels (*P* = .018), higher ESR (*P* < .001) and higher CRP (*P* = .038) levels. It is possible that the regulation of Serpina3 metabolism promotes the EAT inflammatory response associated with HF. Researches on the role of Serpina3 in immune alteration and inflammatory response are needed. Furthermore, HF was shown to be related to the development of cancer.^28^ The presence of failing heart resulted in significantly increased intestinal tumour load, with markedly elevated Serpina3 levels compared with healthy human beings. Serpina3 has been proposed as markers of tumour progression of adenoma into carcinoma and is linked to a variety of cancer.^29^, ^30^ Whether the role of Serpina3 in neoplasia would influence the long‐term prognosis of HF still needs to be investigated.

We were aware of several limitations in our study, which should be taken into consideration. First, the relatively small sample size in this study may weaken the association of candidate EAT proteins with HF. The role of Serpina3 in HF should be further discussed in a large‐scale prospective study. Second, only plasma Serpina3 was measured in the validation phase without other differentially expressed proteins. Plasma‐based screening assay should be applied in the validation studies. Third, there is not enough evidence to illustrate if Serpina3 produced in the EAT might be released into circulation. The source of circulating plasma Serpina3 in humans is unclear. Fourth, we only discussed HF with reduced ejection fraction. Because of the proteomic profile of HF in different stage might be different, the variability amongst patients needs to be considered for sample inclusion. Finally, this discovery phrase is the first step toward the understanding of EAT in response to HF. A study focused on the mechanism will be followed.

In summary, EAT has become research focus regarding its implication in the genesis and progression of HF. By establishing genetic and proteomic networks with proteomic alterations, we described a comprehensive dimension of potential indicators for pathogenesis and therapeutic target of HF. Several proteins were differentially expressed in HF, whilst Serpina3 serves important function in pathways known to be involved in the genesis of HF such as inflammatory response. Although our findings are descriptive, they pinpointed key regulators for the mechanism of EAT in HF and offered fertile grounds for future mechanistic studies.

## CONFLICT OF INTEREST

The authors declare that they have no conflict of interest.

## AUTHOR CONTRIBUTIONS

LZ and XY designed the study. ZG and PW acquired the data. MZ, XY, YL and ZM did the analysis and interpretation of data. LZ and ZG wrote the manuscript. MC and XY revised the manuscript.

## Data Availability

The mass spectrometry proteomics data have been deposited to the ProteomeXchange Consortium via the PRIDE partner repository with the data set identifier PXD014592.
