# Supplementary figures and images for: Proteomics of epicardial adipose tissue in patients with heart failure
Source: J Cell Mol Med. 2019 Oct 31;24(1):511–20. doi: 10.1111/jcmm.14758 (PMC6933327; doi:10.1111/jcmm.14758)

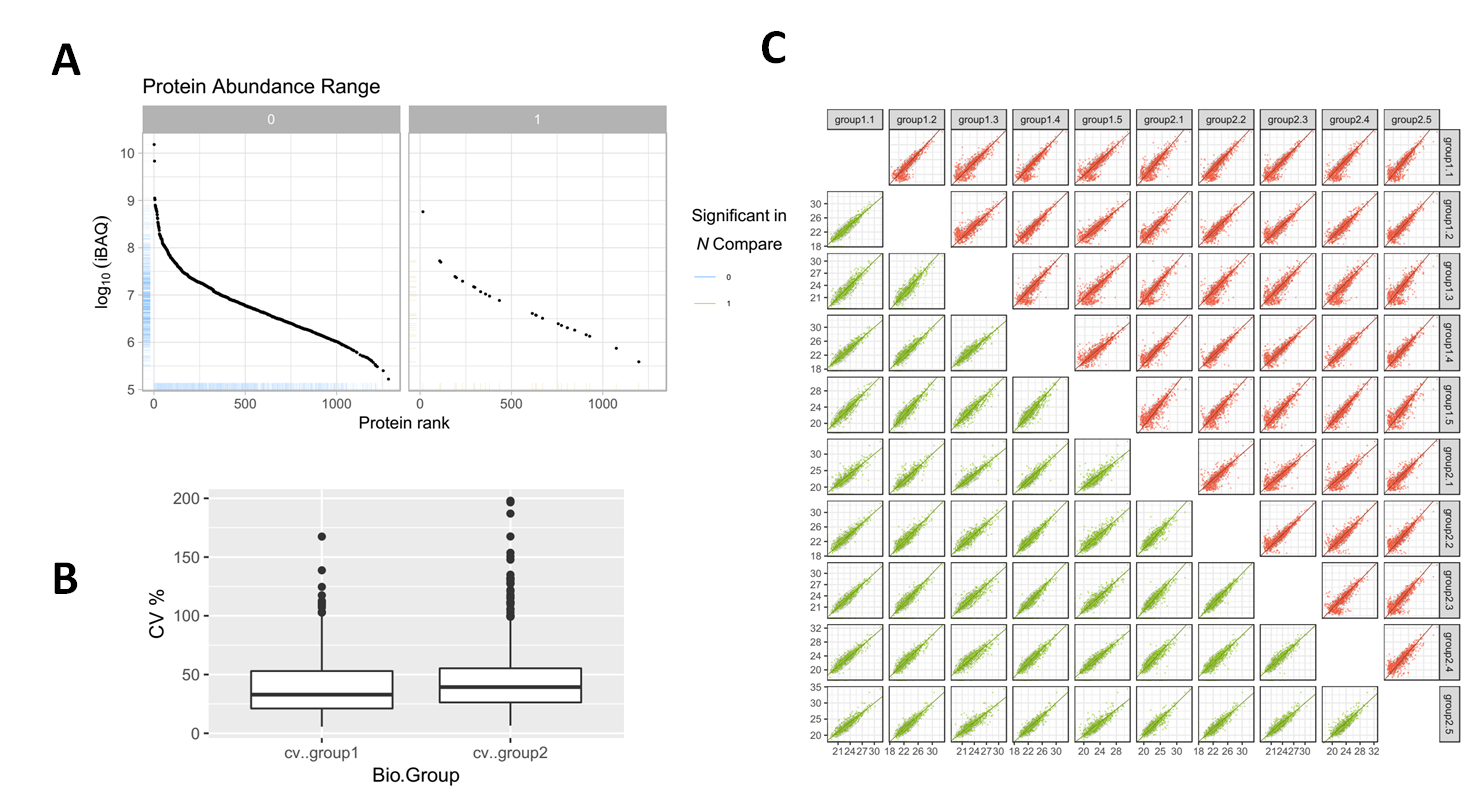

Supplement: Supplementary file 1 [file JCMM-24-511-s001.tif]

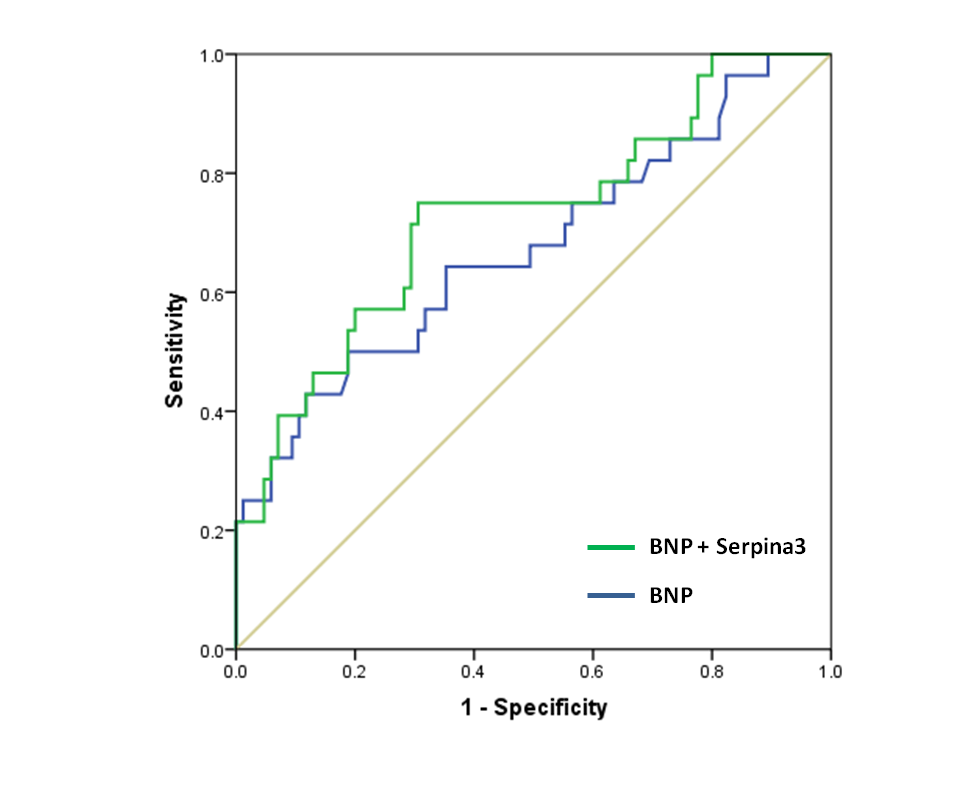

Supplement: Supplementary file 2 [file JCMM-24-511-s002.tif]
