# Supplementary material for: Proteomics of epicardial adipose tissue in patients with heart failure
Source: J Cell Mol Med. 2019 Oct 31;24(1):511–20. doi: 10.1111/jcmm.14758 (PMC6933327; doi:10.1111/jcmm.14758)
Supplement: Supplementary file 3 [file JCMM-24-511-s003.docx]

| Supplementary Table 1. Demographic and clinical characteristics of patients in the proteomics analysis. | | | |
| --- | --- | --- | --- |
| Characteristics | Non-HF (n=5) | HF (n=5) | *p* value |
| Age, years | 61 ± 8 | 68 ± 5 | 0.135 |
| Male, n, % | 4 (80%) | 2 (40%) | 0.524 |
| BMI, kg/m^2^ | 25.04 ± 5.48 | 23.87 ± 4.56 | 0.723 |
| Systolic blood pressure, mmHg | 121 ± 10 | 127 ± 37 | 0.734 |
| Diastolic blood pressure, mmHg | 73 ± 9 | 68 ± 9 | 0.476 |
| Diabetes, n, % | 2 (40%) | 2 (40%) | 1.0 |
| Hypertension, n, % | 5 (100%) | 4 (80%) | 1.0 |
| BNP, ng/L | 89 (52, 145) | 1091 (927, 3366) | 0.008 |
| C-reactive protein, mg/L | 6.86 ± 4.57 | 6.51 ± 6.73 | 0.934 |
| Fast glucose, mmol/L | 8.87 ± 6.56 | 11.76 ± 6.41 | 0.501 |
| Total cholesterol, mmol/L | 3.60 ± 1.53 | 3.67 ± 3.07 | 0.962 |
| LDL, mmol/L | 2.24 ± 1.46 | 2.34 ± 2.52 | 0.941 |
| HDL, mmol/L | 0.7 ± 0.12 | 0.84 ± 0.54 | 0.585 |
| Triglycerides, mmol/L | 1.86 ± 0.89 | 0.89 ± 0.38 | 0.056 |
| Uric acid, μmol/L | 357 ± 69.29 | 363.2 ± 82.37 | 0.901 |
| Serum creatinine, μmol/L | 76.8 ± 16.9 | 88.24 ± 25.22 | 0.424 |
| LVEDD, mm | 47 (43, 48) | 59 (56, 66) | 0.008 |
| LVEF, % | 60 (58, 67) | 45 (35, 49) | 0.008 |
| BMI, body mass index; BNP; brain natriuretic peptide; HDL, high density lipoprotein; HF, heart failure; LDL, low density lipoprotein; LVEDD, left ventricular end diastolic diameter; LVEF, left ventricular ejection fraction. | | | |
