# Supplementary material for: Proteomics of epicardial adipose tissue in patients with heart failure
Source: J Cell Mol Med. 2019 Oct 31;24(1):511–20. doi: 10.1111/jcmm.14758 (PMC6933327; doi:10.1111/jcmm.14758)
Supplement: Supplementary file 4 [file JCMM-24-511-s004.docx]

| Supplementary Table 2. Basic clinical and laboratory characteristics in patients with or without HF. | | | |
| --- | --- | --- | --- |
| Clinical Variables | Non-HF (n=88) | HF (n=28) | *P* value |
| *Demography* |  |  |  |
| Age, years | 63 ± 13 | 60 ± 13 | 0.304 |
| Male, n, % | 62 (70.45%) | 21 (75%) | 0.642 |
| BMI, kg/m^2^ | 25.46 ± 3.07 | 26.17 ± 3.21 | 0.369 |
| Heart rate, bpm | 79 ± 13 | 91 ± 25 | **0.001** |
| Systolic blood pressure, mmHg | 132 ± 19 | 126 ± 24 | 0.207 |
| Diastolic blood pressure, mmHg | 73 ± 13 | 77 ± 12 | 0.167 |
| Current smoker, n, % | 47 (53.41%) | 17 (60.71%) | 0.498 |
| Hypertension, n, % | 48 (54.55%) | 16 (57.14%) | 0.81 |
| Diabetes mellitus, n, % | 30 (34.09%) | 13 (46.43%) | 0.239 |
| Family history of CAD, n, % | 31 (35.23%) | 8 (28.57%) | 0.516 |
| *Laboratory findings* |  |  |  |
| Hemoglobin, g/L | 129 ± 16 | 132 ± 23 | 0.411 |
| Leukocytes, ×10^9^/L | 9.27 ± 2.74 | 10.78 ± 4.0 | **0.026** |
| Neutrophil, ×10^9^/L | 6.83 ± 2.51 | 8.33 ± 3.57 | **0.015** |
| Lymphocyte, ×10^9^/L | 1.67 ± 0.65 | 1.64 ± 0.67 | 0.801 |
| Platelets, ×10^9^/L | 204.65 ± 69.71 | 204.43 ± 75.0 | 0.989 |
| Serum albumin, g/L | 39.4 (37.5, 41.6) | 36.5 (34.8, 41.0) | 0.069 |
| Total Cholesterol, mmol/L | 5.02 ± 1.55 | 4.75 ± 1.06 | 0.399 |
| HDL, mmol/L | 1.06 ± 0.24 | 1.07 ± 0.39 | 0.955 |
| LDL, mmol/L | 2.97 ± 1.03 | 2.87 ± 1.09 | 0.653 |
| Triglycerides, mmol/L | 1.74 ± 1.76 | 1.6 ± 0.94 | 0.703 |
| Troponin-I, ng/mL | 47.59 ± 78.46 | 64.61 ± 84.75 | 0.329 |
| CK-MB, ng/mL | 36.29 ± 63.29 | 67.04 ± 120.91 | 0.232 |
| BNP, pg/mL | 186 (77.5, 401) | 375.5 (112, 1042.25) | **0.006** |
| ESR, mm/h | 14.81 ± 12.29 | 20.87 ± 17.58 | 0.061 |
| C-reactive protein, mg/L | 5.28 (2.3, 13.73) | 13.81 (9.54, 15.44) | **0.001** |
| Serum creatinine, μmol/L | 76.7 (64.7, 95.8) | 85.45 (73.03, 137.18) | **0.015** |
| BUN, mmol/L | 5.64 (4.62, 6.78) | 7.72 (4.63, 9.83) | **0.013** |
| Fast glucose, mmol/L | 7.29 ± 3.29 | 8.26 ± 3.09 | 0.172 |
| Fibrinogen, mg/dL | 293.02 ± 100.89 | 364.0 ± 122.14 | **0.003** |
| *Echocardiography* |  |  |  |
| LAD, mm | 35 ± 4 | 38 ± 4 | **0.01** |
| LVEDD, mm | 47 (44, 49) | 51 (48, 55) | **<0.001** |
| LVESD, mm | 29 (27, 32) | 37 (34, 42) | **<0.001** |
| LVEF, % | 60 (56, 67) | 41 (35, 46) | **<0.001** |
| *Infarct characteristics, n, %* |  |  | 0.288 |
| STEMI | 54 (61.36%) | 14 (50%) |  |
| NSTEMI | 34 (38.64%) | 14 (50%) |  |
| BMI, body mass index; BNP, brain natriuretic peptide; BUN, blood urea nitrogen; CAD, coronary artery disease; CK-MB, creatine kinase MB; ESR, erythrocyte sedimentation rate; HDL, high-density lipoprotein; HF, heart failure; LAD, left atrial diameter ; LDL, low-density lipoprotein; LVEDD, left ventricular end diastolic diameter; LVEF, left ventricular ejection fraction; LVESD, left ventricular end systolic diameter; NSTEMI, non-ST-segment elevation myocardial infarction; STEMI, ST-segment elevation myocardial infarction. | | | |
