# Supplementary material for: Proteomics of epicardial adipose tissue in patients with heart failure
Source: J Cell Mol Med. 2019 Oct 31;24(1):511–20. doi: 10.1111/jcmm.14758 (PMC6933327; doi:10.1111/jcmm.14758)
Supplement: Supplementary file 6 [file JCMM-24-511-s006.docx]

| Supplementary Table 4**.** Univariate and multivariate linear regression models for Serpina3. | | | | | | |
| --- | --- | --- | --- | --- | --- | --- |
| Variable | Univariate Analysis | |  | | Multivariate Analysis | |
|  | Standardized β | P Value |  | | Standardized β | P Value |
| BMI, kg/m^2^ | -0.232 | 0.041 | | -0.243 | | 0.032 |
| Heart rate, bpm | 0.222 | 0.017 | | — | | — |
| BUN, mmol/L | 0.229 | 0.014 | | — | | — |
| BNP, pg/mL | 0.223 | 0.017 | | — | | — |
| ESR, mm/h | 0.363 | <0.001 | | 0.269 | | 0.040 |
| C-reative protein, mg/L | 0.206 | 0.038 | | — | | — |
| Serum albumin, g/L | -0.244 | 0.009 | | — | | — |
| Fibrinogen, mg/dL | 0.379 | <0.001 | | 0.335 | | 0.012 |
| BMI, body mass index; BNP, brain natriuretic peptide; BUN, blood urea nitrogen; ESR, erythrocyte sedimentation rate. | | | | | | |
