# Supplementary material for: Proteomics of epicardial adipose tissue in patients with heart failure
Source: J Cell Mol Med. 2019 Oct 31;24(1):511–20. doi: 10.1111/jcmm.14758 (PMC6933327; doi:10.1111/jcmm.14758)
Supplement: Supplementary file 7 [file JCMM-24-511-s007.docx]

| Supplementary Table 5**.** Univariate and multivariate linear regression models for heart failure. | | | | | | |
| --- | --- | --- | --- | --- | --- | --- |
| Variable | Univariate Analysis | |  | | Multivariate Analysis | |
|  | Standardized β | P Value |  | | Standardized β | P Value |
| Heart rate, bpm | 0.294 | 0.001 | | 0.219 | | 0.014 |
| BNP, pg/mL | 0.398 | <0.001 | | 0.354 | | <0.001 |
| Serpina3, μg/mL | 0.214 | 0.021 | | 0.092 | | 0.303 |
| Fibrinogen, mg/dL | 0.278 | 0.003 | | 0.157 | | 0.093 |
| BNP, brain natriuretic peptide. | | | | | | |
