# Supplementary material for: Proteomics of epicardial adipose tissue in patients with heart failure
Source: J Cell Mol Med. 2019 Oct 31;24(1):511–20. doi: 10.1111/jcmm.14758 (PMC6933327; doi:10.1111/jcmm.14758)
Supplement: Supplementary file 8 [file JCMM-24-511-s008.docx]

**Supplementary Figure legends**

**Supplementary Figure 1.** Quantitative profiling of EAT between HF and non-HF groups. A. Protein abundance ranking by iBAQ value. B. The overall percentage coefficient variation (% CV) across measurements. C. Normalization summary.

**Supplementary Figure 2.** ROC curve analysis for the prediction of heart failure.
